# Anterior choroidal artery and fetal-type posterior communicating artery origin transposition: a case report

**DOI:** 10.3389/fsurg.2025.1690584

**Published:** 2026-01-12

**Authors:** Zhiyong Lu, Lin Ma

**Affiliations:** Seventh Affiliated Hospital, Sun Yat-sen University, Shenzhen, China

**Keywords:** anterior choroidal artery (AChA), case report, ICA, origin transposition, posterior communicating artery

## Abstract

The Anterior Choroidal Artery (AChA) is an important intracranial artery, mainly supplying blood to key structures such as the internal capsule, optic tract, and lateral geniculate body. Typically, the AChA originates from the internal carotid artery (ICA) as the most distal branch, between the site of origin of the posterior communicating artery (PCoA) and the terminal bifurcation of the ICA. But there are occasional anatomical variations. Understanding these anatomical variations is vital for neurosurgical or neurointerventional procedures, as accidental damage to the AChA can lead to serious consequences. This article described fusiform dilation of ICA and the origins of AChA and PCoA from ICA in exchanged positions.

## Introduction

The AChA plays an important role in brain development and the maintenance of key functions, such as motor, sensory, visual, extrapyramidal, memory, and arousal functions (1). Damage to the AChA can result in severe neurological deficits, such as hemiplegia,hemisensory impairment, homonymous hemianopia, decreased level of consciousness, and extrapyramidal symptoms.From an anatomical perspective, the AChA usually arises from the posterolateral wall of the ICA's communicating segment, at a distance of 2–5 mm distal to the PCoA's origin, and runs posteromedially. It crosses over the optic tract and continues in the interpeduncular cistern, entering the choroidal fissure at the level of the knee of the temporal horn, giving off branches along the way to supply important structures such as the posterior limb of the internal capsule, optic tract, and lateral geniculate body (1–3). Studies have found that the AChA is not consistently fixed in terms of origin, size, course, branching, and blood supply range, with various variations existing. Among them, abnormal origin of the AChA is a rare but important anatomical variation (4). Reported variant origins include the proximal segment of the PCoA, the middle cerebral artery (MCA), the bifurcation of ICA, or a shared origin with the PCoA (5). Anatomical variation in which the AChA arises from the ICA proximal to the PCoA's origin is exceedingly rare (2, 6–10). To our knowledge, this constitutes the seventh reported case of this particular origin variant, and the first case associated with a fusiform aneurysm of the ICA at the communicating segment.

## Case report

**Figure 1:**
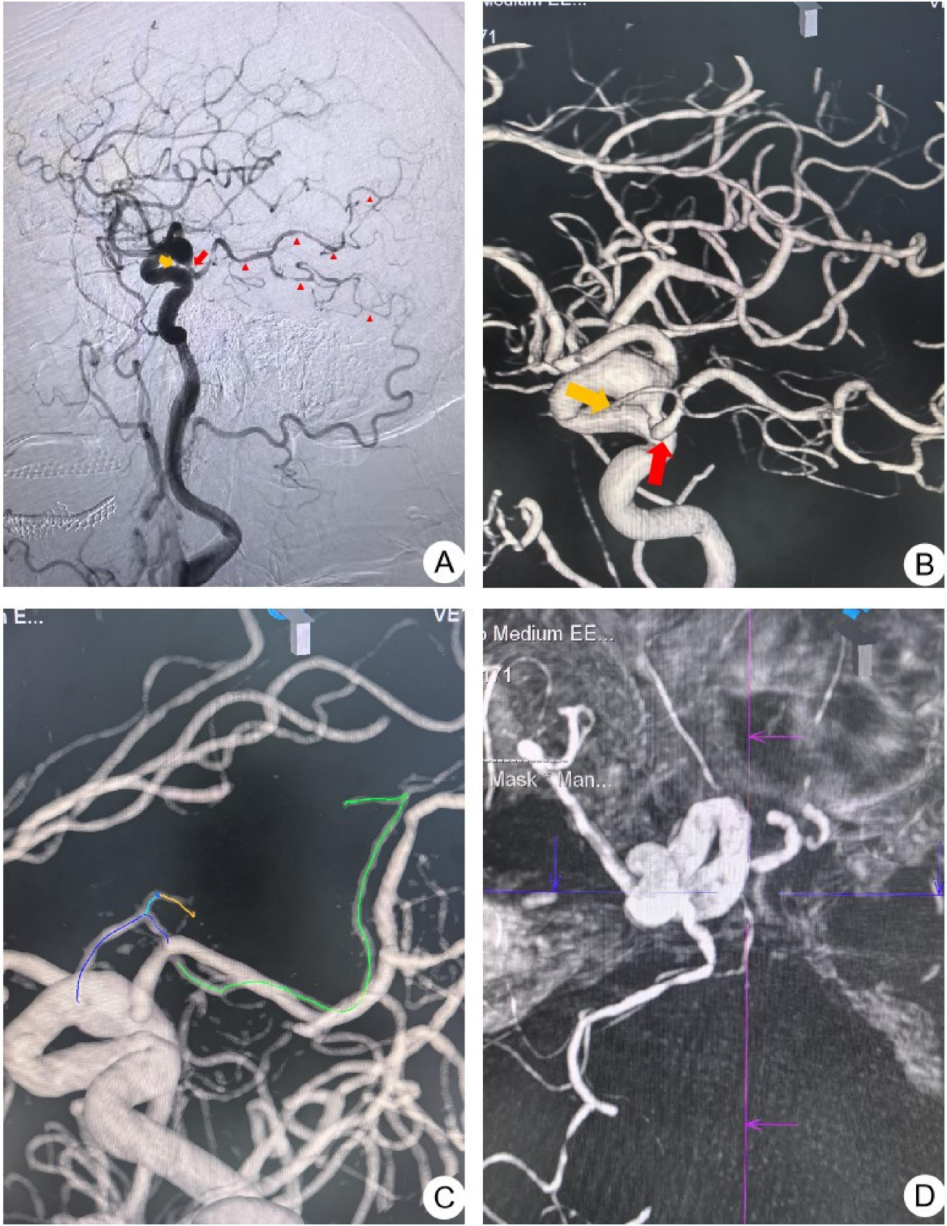
**(A)** Lateral digital subtraction angiography (DSA) of the right ICA shows fusiform dilatation of the C7 segment. The PCoA (red arrow) originates distally and supplies the frontotemporal region, with a small branch (AChA, yellow arrow) arising immediately proximal to its ostium. The area indicated by the red triangle represents the vascular territory of the PCoA. **(B)** Three-dimensional (3D) rotational angiogram delineates the sequential origins of the AChA (yellow arrow) and PCoA (red arrow). **(C)** A computer-automated tracing map depicts the entire course of the AChA: the purple line denotes the proximal segment of the AChA (cisternal segment, portion before entering the choroidal fissure), the green line represents the distal segment (plexal segment, portion after entering the choroidal fissure), and the blue and yellow lines indicate the anterior hippocampal artery. **(D)** Computed tomography angiography (CTA) confirms the origin of the AChA from the proximal segment of the ICA.

A 60-year-old woman visited the clinic due to “recurrent dizziness and headache for 2 months”. She had a history of hypertension for many years. No abnormalities were found in the neurological examination. Outpatient cranial CT angiography (CTA) revealed a fusiform dilation of the intracranial segment of the right ICA. After admission, further cerebral angiography (DSA) revealed a local fusiform dilation of the C7 segment of the right ICA, which was considered to be a fusiform aneurysm. Moreover, the AChA and the fetal-type PCoA originated from the aneurysm. Further observation found that AChA supplying the internal capsule region originate from the relatively proximal end, while the PCoA that perfuse the posterior circulation arise from its distal end, which was considered to be a transposition of the origin of the AChA and the fetal-type PCoA. We observed that there was no anatomical variation in the origins of the contralateral PCoA and AChA in this patient (see Figure 1). The patient was advised to undergo endovascular treatment with a flow-diverter stent for the aneurysm. However, the patient refused the surgery after understanding the surgical risks.

## Discussion

The transposition of the origin of the AChA and the PCoA is an extremely rare anatomical variation of the cerebral vasculature, with only a few case reports documented in the literature (2, 6–10). This anatomical variation may gradually form during the embryonic development process. Embryologically, the primitive ICA divides into two plexiform segments, the rostral and the caudal. The AChA develops from the rostral segment, together with the MCA plexus, and primitive olfactory artery, which median part represents the future anterior cerebral artery(ACA), interconnected by a vascular plexus of future anterior communicating artery (ACoA) (7). The blood flows in the rostrocaudal direction. However, due to extreme growth of the prosencephalon the AChA moves dorsaly and caudaly from its rostral extension, maintaining the postnatal direction (11). Normal embryonal development explains that arterial variations in the brain are formed by modifications of the primary capillary pattern by means of progression and differentiation of one group or regression of another group of capillaries depending on the hemodynamic predominance of the feeding arteries depending on the development of the brain. At Padget stage 5 (intermediate embryonic period), a precocious “capture” of the territory normally destined for the AChA by the PCoA—via competitive capillary regression and expansion—effects an identity swap between the embryonic caudal and rostral branches, thereby precipitating the postnatal origin transposition (12, 13). To date, six cases of this type of AChA origin variation have been reported (Table 1), four of which were associated with aneurysms in different locations (one involving the AChA origin, two involving the PCoA origin, and one involving the C6 segment of the ICA). This case is the seventh reported instance but the first to describe a transposition of the origin of the AChA and the fetal-type PCoA in conjunction with a fusiform aneurysm of the ICA.

**Table 1:** Case reports describing an anomalous origin of the anterior choroidal artery.

Case	Authors, Year	Sex/Age	Symptoms	Associated disease
1	Hara et al., 1989	32/Man	Facial pain	No related diseases
2	Moyer et al., 1992	36/Woman	Sudden loss of consciousness	Multiple dispersed aneurysms, including an aneurysm near the origin of the left PCoA, and SAH caused by aneurysm rupture
3	Nomura et al., 2000	57/Woman	Headache	Unruptured aneurysm at the origin of the left AChA
4	Nishio et al., 2009	56/woman	Dizziness	Unruptured aneurysm at the origin of the left AChA
5	Choi et al., 2012	40/Woman	Sudden headache	Multiple dispersed aneurysms and SAH due to aneurysm rupture caused by the co—sided fetal—type PCoA
6	Mamaliga et al., 2019	62/Woman	Examination after a fall	No related diseases

PCoA, posterior communicating artery; SAH, subarachnoid hemorrhage; AChA, anterior choroidal artery (2, 6–10).

Currently, flow-diverter stent placement is the preferred treatment for intracranial fusiform aneurysms. Maintaining the patency of critical vascular branches, particularly the AChA, is essential to minimize postoperative disability. In this case, flow-diverter stent placement was planned; however, the preoperative identification of the transposition of the AChA and PCoA origins required caution to avoid excessive stent density at the AChA origin, which could compromise AChA blood flow. Although the patient ultimately declined surgery, this case highlights the importance of clarifying the origin of the AChA before surgical or endovascular interventions to prevent inadvertent damage. Given the critical territory supplied by the AChA and the limited collateral circulation, injury during surgery or endovascular treatment may lead to cerebral infarction, resulting in symptoms such as hemiplegia, hemisensory deficits, and homonymous hemianopia. Hyun et al. reported a case of an AChA origin aneurysm treated with coiling; despite maintaining AChA patency, the patient developed an acute infarct in the internal capsule (1). Nomura et al. described a case of AChA and PCoA origin transposition with an aneurysm at the AChA origin. During attempted coiling, the indistinguishable neck between the aneurysm and the AChA origin led to a high risk of AChA occlusion, ultimately resulting in abandonment of the coiling procedure (4). Misidentifying the AChA as the PCoA for coiling could lead to severe neurological deficits.

Patients with ectopic origin of the AChA may present with subarachnoid hemorrhage due to aneurysm rupture or may be incidentally discovered during evaluation for other conditions. These symptoms may be related to the vascular anomaly itself or to the associated aneurysm. Moyer et al. reported a patient with subarachnoid hemorrhage caused by aneurysm rupture, with angiography showing that the AChA originated from the proximal end of the PCoA and an aneurysm at the PCoA origin, which was successfully treated with endovascular coiling (9). Nishio et al. described a patient who presented with dizziness and was found to have a left ICA aneurysm on brain MRA. Angiography revealed that the AChA originated from the proximal end of the PCoA, with the ICA aneurysm located proximal to the AChA. The aneurysm was treated with coiling while ensuring patency of the AChA (8).

When the AChA and PCoA have similar calibers, such as a slender PCoA or a hypertrophied AChA, distinguishing between them can be challenging in the presence of an ectopic origin variation. This may lead to potential misdiagnosis or oversight. Angiography is a key diagnostic tool for identifying ectopic origin of the AChA, including both conventional angiography and three-dimensional rotational angiography (3D-RA). Three-dimensional imaging has significant advantages in visualizing the spatial relationships between vessels such as the AChA and PCoA, aiding in accurate determination of vessel origins and courses. Additionally, maximum intensity projection (MIP) reconstructions in the axial and sagittal planes from CT angiography can help assess vessel courses and perfusion territories (6). Nishio et al. used 3D-RA to clarify the relationship between the AChA and PCoA, highlighting the importance of three-dimensional imaging in diagnosing such anomalies (8).

## Conclusion

The abnormal origin of the AChA is an important anatomical variation that neurosurgeons and neurointerventionalists should fully recognize before surgery to avoid catastrophic consequences due to intraoperative damage. If such vascular anomalies are detected on preoperative cranial MRA or CTA, further assessment with cerebral angiography (DSA) is essential, and a meticulous surgical plan should be formulated to minimize the risk of complications. More research is needed in the future to explore the embryological mechanisms and clinical strategies for dealing with such anomalies, thereby improving the level of diagnosis and treatment.

## Data Availability

The raw data supporting the conclusions of this article will be made available by the authors, without undue reservation.
